# Retraction: Ribosomal L22-like1 (*RPL22L1*) Promotes Ovarian Cancer Metastasis by Inducing Epithelial-to-Mesenchymal Transition

**DOI:** 10.1371/journal.pone.0342378

**Published:** 2026-02-09

**Authors:** The PLOS One Editors

After this article [1] was published, concerns were raised about Fig 4, and subsequent editorial assessment identified additional issues. Specifically,

- Panel 8910-RPL22L1 in Fig 4G appears similar to panel 8910PM-RPL22L1 in Fig 4H. The corresponding author stated that the unintentional image duplication was a result of an error in the figure assembly process, and they provided underlying data for Figs 4G and 4H; however, the *PLOS One* Editors consider that concerns about the reliability of the data and results were not fully resolved.
- Two of the four cell lines used in this study [1] are reported as misclassified. Specifically, this article [1] presents both HO8910 and HO8910PM as a ‘human ovarian cancer cell line’ and studies them in the context of ovarian cancer metastasis. However, the HO8910 and HO8910PM cell lines have been reported as misidentified or contaminated, and shown to be derived from HeLa cells (see ICLAC Register of Misidentified Cell Lines (iclac.org/databases/cross-contaminations/) [2], Cellosaurus (https://web.expasy.org/cellosaurus/) [3], and [4,5]). The corresponding author stated that they consider the use of HO8910 and HO8910PM cell lines does not invalidate the gene-centric mechanistic findings of the study. They submitted STR profiling documentation for the HO8910 and HO8910PM cell lines used in the study, which the *PLOS One* Editors consider did not resolve the concern; however, it is noted that the study included two other cell lines for which no identification issues have been reported.
- The *PLOS One* Editors identified concerns about the article’s peer review. We regret that the issues were not addressed before the article’s publication.

In light of the cumulative concerns, which call into question the reliability of the reported results, the *PLOS One* Editors retract this article.

The *PLOS One* Editors additionally note that the quantitative data underlying the results reported for the experiments in cell lines have not been provided within the paper or its Supporting Information files, as indicated in the Data Availability statement. As such, the article does not appear to meet the requirements of the Data Availability policy in place at the time of submission. However, the corresponding author indicated on follow-up that the complete underlying quantitative data could be provided; as such, PLOS considers that this issue potentially could have been resolved, and it did not contribute to the final decision to retract.

NW, JW, YW, JY, YQ, BP, DS, HS, YY, WS, XM, CZ, JB, FC, SF, and YJ did not agree with the retraction and stand by the article’s findings. DT, JG, and KYL either did not respond directly or could not be reached.

## References

[1] Wu N, Wei J, Wang Y, Yan J, Qin Y, Tong D, et al. RETRACTED: Ribosomal L22-like1 (*RPL22L1*) Promotes Ovarian Cancer Metastasis by Inducing Epithelial-to-Mesenchymal Transition. PLoS One. 2015;10(11):e0143659. doi: 10.1371/journal.pone.0143659 26618703 PMC4664398

[2] Capes-Davis A, Theodosopoulos G, Atkin I, Drexler HG, Kohara A, MacLeod RAF, et al. Check your cultures! A list of cross-contaminated or misidentified cell lines. Int J Cancer. 2010;127(1):1–8. doi: 10.1002/ijc.25242 20143388

[3] Bairoch A. The Cellosaurus, a Cell-Line Knowledge Resource. J Biomol Tech. 2018;29(2):25–38. doi: 10.7171/jbt.18-2902-002 29805321 PMC5945021

[4] Ye F, Chen C, Qin J, Liu J, Zheng C. Genetic profiling reveals an alarming rate of cross-contamination among human cell lines used in China. FASEB J. 2015;29(10):4268–72. doi: 10.1096/fj.14-266718 26116706

[5] Bian X, Yang Z, Feng H, Sun H, Liu Y. A Combination of Species Identification and STR Profiling Identifies Cross-contaminated Cells from 482 Human Tumor Cell Lines. Sci Rep. 2017;7(1):9774. doi: 10.1038/s41598-017-09660-w 28851942 PMC5575032

